# Beyond Locks and Lectures: What Rural Parents Think Would Be Effective Firearm Safety Messaging and Programming

**DOI:** 10.1007/s10900-025-01533-y

**Published:** 2025-11-27

**Authors:** Victor A. Soupene, Charles Jennissen, Nicholas Stange, Pam Hoogerwerf, Cassidy Branch, Marc Doobay

**Affiliations:** 1 Department of Emergency Medicine, University of Iowa Health Care, Iowa City, IA USA; 2 Department of Occupational and Environmental Health, University of Iowa College of Public Health, Iowa City, IA USA; 3 Stead Family Department of Pediatrics, Roy J. and Lucille A. Carver College of Medicine, University of Iowa, Iowa City, IA USA; 4 Health Sciences Center School of Medicine, Louisiana State University, Shreveport, LA USA; 5 Injury Prevention and Community Outreach, University of Iowa Stead Family Children’s Hospital, University of Iowa, Iowa City, IA USA; 6 Center for Social Science Innovation, University of Iowa, Iowa City, IA USA; 7 Department of Physician Assistant Studies and Services, Roy J. and Lucille A. Carver College of Medicine, University of Iowa, Iowa City, IA USA; 8 Department of Family and Community Medicine, University of Iowa Health Care, Iowa City, IA USA; 9 Department of Emergency Medicine , University of Iowa Hospitals and Clinics, 200 Hawkins Drive, Iowa City, IA 52242 USA

**Keywords:** Firearms, Health communication, Program implementation, Rural health, Safe storage

## Abstract

Firearm-related suicides and unintentional injuries occur at higher rates in rural U.S. communities than in urban areas. While safe storage programs may help reduce these injuries, little is known about how to effectively engage rural, farming populations. This study aimed to identify factors influencing firearm safety programming and messaging among parents in rural households. Focus groups were conducted in 2024 with rural Iowa parents recruited via FFA club advisors and through the University of Iowa mass e-mail system. Inclusion criteria included: (1) residence in Iowa with active farming or ranching operations, (2) at least one child aged 10–19 living in the home, and (3) at least one firearm in the household. Discussions followed a moderator guide to identify primary themes, with sub-themes emerging organically. Transcripts were analyzed using Dedoose software. Three researchers independently coded the data and resolved discrepancies through consensus. Thirty-two participants identified trusted messengers as law enforcement, Department of Natural Resources personnel, and community members affected by firearm violence. Teachers, healthcare professionals, and celebrities were viewed as less credible. Participants emphasized age-specific messaging using realistic examples and visuals and suggested integrating firearm safety into existing programs like school curricula, 4-H, FFA and local safety courses. Views on distributing safety devices like locks and safes were mixed. Tailoring firearm safety interventions to reflect the values and preferences of rural communities may improve engagement and effectiveness. Strategies led by trusted local messengers may be particularly impactful.

## Introduction

Firearm-related injuries are a major public health concern in the United States (U.S.), contributing to more than 46,000 deaths in 2023 [1]. Age-adjusted U.S. firearm mortality rates were higher among rural populations (17.6 per 100,000) than urban populations (13.1 per 100,000) [1]. Rural areas also experienced higher rates of firearm suicides (11.9 per 100,000 compared to 7.0 per 100,000) and unintentional firearm injuries (0.3 per 100,000 compared to 0.1 per 100,000) relative to their urban counterparts. In contrast, firearm homicide rates were lower in rural areas (4.5 per 100,000) than in urban areas (5.5 per 100,000) [1]. Notably, individuals working in agriculture—who predominantly reside in rural regions—face particularly elevated risks of firearm suicide [2]. These disparities highlight the urgent need for targeted prevention programs to reduce firearm-related injuries and deaths.

Safe firearm storage—defined as keeping firearms locked, unloaded, and stored separately from ammunition—is a proven strategy for reducing unauthorized access and preventing firearm-related injuries [3–5]. Despite its effectiveness, rural populations are less likely to practice safe storage [6, 7], especially among farm households [7–11]. One study found that while the prevalence of loaded firearms in homes was similar between rural and urban firearm owners, rural adults were significantly more likely to store these loaded firearms unlocked [6]. Another study focusing on adolescents in FFA (formerly Future Farmers of America) revealed that those living on farms were more likely to have at least one firearm at home stored either loaded and/or unlocked compared to their peers living in towns [9]. These findings underscore the urgent need to promote and support safe firearm storage practices in rural communities across the U.S.

Rural communities [12–17], especially farming communities [18], are often difficult to engage in research efforts [18]. Several barriers contribute to this challenge, including geographic isolation [12] and a general distrust of outsiders [13]. These difficulties are further compounded by deeply rooted cultural norms and attitudes toward firearms. In many rural areas, firearms are considered “a way of life,” commonly used for social activities such as hunting and sport shooting, as well as for personal protection [7, 14, 19]. Efforts to reduce firearm access in these communities often face resistance due to strong concerns about firearm rights and distrust towards outsiders in their community [16], which hinders the effectiveness of firearm safety messaging and programming [17].

It is important to recognize that rural populations are not monolithic [20]. Significant differences exist among individuals living in rural areas. For instance, those residing on farms or within farming communities often hold distinct views on firearms and safe firearm storage compared to other rural residents [21, 22]. To effectively engage these communities, it is essential to understand the most appropriate methods for delivering firearm safety messaging and programming tailored to rural, farming households. Thus, this study aimed to address the following research questions:

1. Who are trusted messengers for safe firearm storage programming?
2. Who are less trusted messengers for safe firearm storage programming?
3. How should firearm safety messages be delivered to be most effective?
4. What messages and types of programming are most effective for promoting safe firearm storage?

Given the elevated rates of firearm-related injuries—particularly suicides and unintentional injuries—in rural areas, along with higher firearm ownership and lower rates of safe storage, it is critical to explore how to design and deliver effective firearm safety interventions. Identifying key themes related to messaging and programming will help guide the development and dissemination of safe firearm storage strategies tailored to rural firearm-owning adults.

## Methods

###  Study Participants and Recruitment

Focus groups were conducted with eligible participants who met the following criteria: (1) be a parent with at least one child 10–19 years of age; (2) currently live on and/or actively farm or ranch in Iowa; (3) have at least one firearm in the home; and (4) be English-speaking. Only one parent per household was permitted to participate. The study was approved by the University of Iowa (UI) Institutional Review Board (IRB #202305100). Support for research services (i.e., recruitment, focus group moderation, transcription, and qualitative coding) was provided by the UI Center for Social Science Innovation (CSSI).

Participants were recruited through several methods. Initially, over 230 Iowa FFA chapter advisors were contacted and invited to share the opportunity with parents of their membership and with farming/ranching parents in their school districts. Three advisors assisted in holding in-person focus group sessions of parents at their schools. Each FFA chapter was provided a $500 incentive for hosting the event.

In order to reach study goals, additional subjects were recruited through an email distributed through the internal UI mass email system. The study registration link was visited by 324 individuals, and the CSSI assisted in verifying eligibility using registrants’ mailing addresses and google maps satellite imaging. The CSSI determined that 153 registrants were ineligible, and 118 registrants gave inconclusive contact information. The 53 registrants that appeared to be eligible were stratified into groups according to their focus group session date preferences. A personalized invitation email with a Qualtrics pre-focus group web survey link was sent to registrants approximately 5 days prior to their scheduled session. Upon completion of the web survey, a follow-up email was sent to registrants containing details about the time of the focus group and a Zoom link. The study team also sent a calendar invitation to registrants the day before each on-line focus group session and a reminder e-mail was sent to non-respondents.

### Data Collection

A moderator script was developed by the research team with consultation from the CSSI. After an introduction with a statement of focus group ground rules, which included the promotion of keeping responses confidential within the group, a variety of firearm storage and safety topics were discussed. These included current firearm and ammunition storage practices in the home, best messaging and methods to access and educate families on safe storage, and best individuals or groups to deliver messages or education on safe storage. The moderator guide included a primary question for each topic area as well as 1–5 question prompts to help guide the conversation and explore topics in more detail. Most topic areas were allotted approximately ten minutes for discussion with variations in duration as needed.

Participants provided written (in-person) or electronic (virtual) consent. Before the discussion, they completed a brief online or paper survey assessing (1) demographics (2) firearm safety education, use and storage, and (3) attitudes towards several firearm topics. Focus group sessions were conducted in English, recorded, and approximately ninety minutes in duration. Eight focus groups were completed, and participants received a $100 gift card as compensation for their time.

Audio files were transcribed by the CSSI. First, the audio data was processed using Whisper by OpenAI. Whisper is an automatic speech recognition (ASR) system trained on 680,000 h of multilingual and multitask supervised data collected from the web. Following processing by Whisper, CSSI staff performed manual transcription using Olympus software to correct automated errors. A three-step process was utilized where a staff member reviewed the transcripts and corrected inconsistencies, a second staff member subsequently reviewed the file and performed additional edits for quality, and then a supervisor conducted a read-through with final approval.

### Analysis

Descriptive statistics were utilized to express demographic characteristics of focus group participants. Transcripts were reviewed and coded by the moderator and co-moderator, independently, using Dedoose 9.2.12 software. Coding results and statements were exported as an Excel spreadsheet. This was provided to three research team members who independently coded the transcript statements. Coding was reviewed and discrepancies were reconciled through an iterative process of group discussion to reach consensus. Codes were compiled to address our research questions and reviewed by the full study team.

### Reflexivity

Given our positionality as researchers and clinicians [23], we believe it is important to provide context about our study team and our involvement in the qualitative research process. The research team included clinicians, public health professionals, and qualitative researchers with experience in rural health and firearm safety. Our diverse professional backgrounds informed both data collection and analysis while reinforcing reflexivity and attentiveness to positionality.

## Results

### Descriptive Statistics

A total of 32 individuals participated in the study; 19 participants took part in three in-person focus groups and 13 participants were in 5 virtual sessions. Participants ranged in age from 24 to 60 years, with an average age of 45 years (Table 1). All participants identified as non-Hispanic White, and nearly three-fifths were female. Educational attainment varied, with most participants having post-high school education (68.8%).

**Table 1:** Descriptive characteristics of the study sample (*N* = 32)

Characteristic	*N* (%)
*Sex*	
Male	13 (40.6)
Female	19 (59.4)
*Race/Ethnicity*	
Non-Hispanic White	32 (100.0)
*Level of education*	
High school diploma or less	10 (31.2)
Some college/associate’s degree	11 (34.4)
Bachelor’s degree or more	11 (34.4)
Characteristic	Mean (Standard Deviation, Interquartile Range)
Age	45.5 (5.3, 42.0–49.0)
Long guns at home	11.2 (17.9, 2.0–13.8.0.8)
Handguns at home	3.8 (4.8, 1.0–6.0)

### Qualitative Analyses

All codes are available with examples in Table 2. Codes were organized based on our a priori research questions.

**Table 2:** Summary of study domains, codes, definitions, and boundaries

Overarching research question	Code	Definition (What it is)	Boundary (What it is not)
*Who are trusted messengers for safe firearm safety programming?*	Firearm dealers and safety instructors	Messengers with specialized knowledge or training in firearms, such as hunter safety instructors, firearms dealers, or certified safety trainers.	This does not include individuals who work for these entities without expertise in firearms or firearm safety.
	Law enforcement	Messengers who work in law enforcement, including police officers and sheriffs/deputies.	This may not include other emergency responders like firefighters or any other protective service workers.
	Department of natural resources	Messengers who work for a state Department of Natural Resources.	This does not include workers in any other federal or state government agencies.
	Those personally affected	Messengers who are parents or family members who have experienced a firearm-related injury or death.	This does not include individuals who were exposed to firearm violence through media.
	Community leaders	Messengers recognized as leaders within a specific community.	This does not include federal or state leaders.
*Who are less trusted messengers for safe firearm safety programming?*	Healthcare workers	Messengers who are healthcare professionals, including clinicians and nurses, who discuss firearm safety with their patients.	This does not include other types of healthcare workers or those who promote firearm safety outside of work.
	Educators	Messengers who educate the public through elementary, secondary, or post-secondary schools.	This does not include educators outside of elementary, secondary, or post-secondary schools.
	Politically backed sources	Messengers who are politicians or affiliated with politically recognized groups or organizations.	This does not include community leaders or sources where their political stance is unclear.
	Celebrities	Messengers who are widely recognized public figures or celebrities.	This does not include individuals who are well-known only within the community.
*How should firearm safety messages be delivered to be most effective?*	Television advertisements	Firearm safety messages delivered through television advertisements.	This does not include advertisements shown online.
	Social media	Use of social media, including YouTube and Facebook, for firearm safety messaging.	This does not include traditional media or text messaging.
	Flyers at point of purchase	Physical flyers distributed at the point of purchase, such as with firearms, safes, or hunting licenses.	This does not include physical flyers distributed at other locations.
	FFA and 4-H	Firearm safety booths or shooting sport activities presented at FFA and 4-H-sponsored events.	This does not include other agricultural or child-oriented organizations.
	Agricultural extension offices	Emails and newsletters distributed by sources such as agricultural extension offices.	This does not include emails or newsletters from other sources.
	Schools	Programs delivered in schools, especially through safety events or in coordination with FFA or 4-H.	This does not include programs for schools that are not elementary, secondary, or post-secondary.
*What messages and types of programming are most effective for promoting safe firearm storage?*	Use of personal stories/real-life examples	Personal stories from individuals who have experienced firearm-related tragedies used in firearm safety messaging.	This does not include hypothetical stories or stories from secondary sources.
	Use of visual aids	Visual aids used in firearm safety messaging.	This does not include figures and diagrams of statistics.
	Use of statistics	Inclusion of firearm injury and safety statistics in firearm safety messaging.	This does not include statistics unrelated to firearm violence or safety, or visual representations like graphs.
	Repetition of messaging	Repetition of firearm safety messages to reinforce key points.	This does not include messages that are similar but different when distributed.
	Incentives	Incentives such as free or discounted safety devices (e.g., lockboxes or safes) or promotional events (e.g., raffles)	This does not include other incentives such as monetary rewards.

### Who Are Trusted Messengers for Safe Firearm Safety programming?

Our focus groups identified firearm experts as trusted messengers for the delivery of firearm safety programming. Those considered experts were individuals who are firearm dealers or safety instructors with “*training in weapons*” and were “*professionals that could be a part of [the] solution [for] the problems that are out there with guns.*” Law enforcement officers - specifically “*local law enforcement*” - were the favored group to be distributing firearm safety information. One participant said, “*if you’re talking about rural Iowa specifically*,* I think Sheriff’s Department seems like most rural Iowans respect.*” Some suggested that law enforcement could offer some form of firearm safety education: “*I feel like they’re more there to help to serve in more of that education standpoint than just punitive.*” Many also considered the Department of Natural Resources (DNR) to be a source of reliable information but with a few dissenting opinions, such as “*the DNR*,* I don’t trust them…they’re not fun to get along with sometimes. And I deal with [them]*,* on a daily basis.*”

Individuals who were already affected by firearm violence or “*a person that has lived through a situation*” were also considered trusted sources for providing firearm safety messaging. One participant suggested, “*…like someone that’s been impacted themselves*,* like a parent or child that [has] maybe went through a recovery.*” Some suggested community organizations or a person familiar with the community: “*I wouldn’t take any message from the outside anywhere. So*,* it [would have to] be [someone] close to me.*”

### Who Are Less Trusted Messengers for Safe Firearm Safety programming?

Groups who were not considered “trusted sources” for firearm safety were also identified. Many participants felt disdain regarding healthcare workers and university-associated staff discussing firearm safety with them. One participant said, “*It’s just a doctor*,* I’m getting treated for a nose cold or something like that and they ask*,* tell me about firearm safety*,* I’m not going to trust them…*” Another participant said, “*…it’s not the University. We’re not going to trust them.*” Teachers were also not considered to be trusted sources for safe storage and firearm safety: “*How would you get me to believe that a teacher pushing the message is going to be effective?*” Politically backed sources were also generally considered less trusted, “*…those messages have not been effective because they’ve all been politically motivated.*” Celebrities were mostly not considered reliable sources for firearm safety: “*I think that if [an] actress got on a commercial and said something… it would just go in one ear and out the other.*”

### How Should Firearm Safety Messages Be Delivered To Be Most effective?

Participants suggested several methods regarding how firearm safety messages should be delivered. Traditional media, including television news advertisements, were considered effective for promoting firearm safety messages, especially when the news station is local: “*…news ads on local TV…[just] like… the University has ads about health care services or new clinics.*” Participants also mentioned that television advertisements are effective when the audience is older (“*…really target age demographic and if it’s parents*,* then I think you could do TV shows or regular TV programming*”) but not when targeting young people (“*High school kids*,* it’s an insult if they ever tell you to go watch TV. That’s a major insult that they’ve come up with because they don’t get their information from TV shows anymore. Kids don’t have a favorite TV show anymore - it’s just YouTubers…*”).

Conversely, social media was considered effective regardless of age: “*…there’s all sorts of social media posts that can grab your attention really quick.*” Another participant stated, “*Well*,* unfortunately*,* social media is such a huge thing. Email*,* videos*,* sharing stories*,* horror stories of when people did* *not* *store [firearms] appropriately*,* and what can happen.*” Written handouts and flyers were also seen as effective whether through mailers (“*…like if it was something like a mailer or postcard or something that you’re getting like annually. Sometimes just that reminder of*,* oh yeah like that’s something we should do wouldn’t necessarily be a bad thing*”) or a flyer provided at the point of purchase (“*…part of like purchasing a gun*,* like here’s your flyer about storage.*”).

Participants wanted to see firearm safety programming integrated into existing programs through agricultural-related organizations like FFA and 4-H (“*…come join us and help us raise money for the school in the long run. We can do an auction like [what] we did with the FFA. They raised money for FFA*,* but part of it is talking about gun safety. Like*,* put on something. An event.*”) or emails or newsletters from agricultural extensions (“*…every county is covered by an extension service; every county in Iowa is covered. And I would think*,* somehow*,* through them*,* there would be a way to identify all rural homes*”). Some participants suggested firearm safety programs already exist but could probably be expanded in schools: “*Gun safety is already part of elementary [school]; it’s very brief*,* but in a public school*,* that’s already part of their curriculum.*”

### What Messages and Types of Programming Are Most Effective for Promoting Safe Firearm storage?

Participants suggested that messaging should use personal stories and “real-life” examples: “*I like personal stories.*” Several participants felt personal stories were impactful (”*…have [their] family come in and share their story*,* their side*,* like how it made them feel*”). Another participant stated, “*I’m always kind of moved by like real-life examples.*” Messaging should also include graphic visuals: “*…the parents should probably have [a] visual aid. Like what? Scare the [expletive] out of them.*”

There were mixed feelings towards using statistics in firearm safety messaging: “*Hard data would make me start thinking about [changing storage practices].*” Another participant stated, “*I’ll read through the statistics*,* but it might not persuade the way I store my guns. It might have an effect*,* more so as like*,* ‘Oh*,* that’s a better storage option*,*’ or ‘That’s a good idea.’ More different ideas and how to store would be more persuasive to me than statistics would be.*”

While some participants voiced the importance of repeating firearm safety messages, others had reservations regarding repeat firearm safety requirements: “*I think everyone needs repetition for most things that they’re learning*,* or that are important. I know I do.*” However, another participant stated, “*Implementing something where every two years or five years*,* you got to repeat something*,* I don’t see any value in that.*”

Participants had conflicting opinions on the effectiveness of distributing free or reduced cost firearm safety devices. For firearm safety locks, some participants suggested, “*I don’t know who would say no to the safety if it’s offered to them*” or “*it’s certainly better than nothing… if you are willing to use them.*” Others said the firearm locks would not be used: “*We’ve probably picked up more free gun locks over the years than I can shake a stick at*,* and I don’t think we’ve ever used one.*”

More participants considered offering free or reduced-cost firearm safes to be much more effective: “*…if somebody wanted to provide me another lock box so I [could] put the ammunition in it*,* I don’t have a couple grand to drop on another lock box like currently.*” Some participants suggested hosting a lottery event: “*Let’s have a raffle for a drawing*,* for a gun safe… like a door [prize]….*” However, some participants believed providing free or reduced-cost safety devices in any capacity were ineffective: “*I don’t think incentives work*,* I think it’s all about education*” or “*There are incentives that work for different things*,* but I don’t think this is one of them.*”

## Discussion

Firearm safety messaging and programming among rural adults is complex and shaped by several key themes. One consistent finding is the importance of the messenger. Rural adults tend to place greater trust in individuals who are local and perceived to have firsthand experience with firearms—such as law enforcement officers and personnel from the DNR. This finding is consistent with previous research that law enforcement personnel were seen as more credible when discussing safe firearm storage [14]. This highlights the importance of tailoring firearm safety interventions to align with the values and trusted voices within rural communities. Future efforts to develop and implement firearm safety programs should carefully consider who delivers the message, as the credibility of the messenger can significantly influence both the reach and effectiveness of the intervention.

Conversely, clinicians and educators were perceived as less trustworthy. This finding aligns with previous research on rural perspectives regarding firearm storage, which indicated that clinicians and educators are often viewed as less credible messengers for firearm safety. One possible explanation is the rise of anti-intellectualism, which may contribute to skepticism toward these professions [24]. An ethnographic study of rural residents in Wisconsin suggested that individuals in rural areas often feel stigmatized by those from urban settings, particularly those affiliated with academic institutions [25]. Additionally, longstanding disparities in access to higher education between rural and urban populations—where rural communities are often disadvantaged—may further erode trust in physicians and educators [20, 26]. It is important to recognize these contextual factors within rural populations and aim to rebuild trust through approaches such as community-based participatory research and stakeholder engagement [20].

This study also identified several factors that influence the effectiveness of firearm safety strategies, with age emerging as a key consideration. Prior research has shown that age plays a vital role in shaping how individuals prefer to receive health and safety information. For example, one study found that social media is a preferred platform for disseminating such information, particularly among younger populations [27]. In addition, organizations that already provide firearm safety education and programming—especially those trusted by children and adolescents—should be considered valuable partners for distributing additional safety materials. Leveraging these trusted sources may enhance the reach and impact of firearm safety messaging across different age groups. Overall, participants described stories and practical examples as more compelling than statistics alone.

Participants expressed mixed views on the effectiveness of distributing firearm safety devices. This finding differs from previous research [28, 29] demonstrating a preference among most firearm owners for the distribution of free or reduced-cost firearm safety devices. While existing research supports the idea that providing free or low-cost firearm locks and safes can lead to safer storage practices [29–32], our findings suggest that the success of such interventions may depend on how they are delivered. Messenger credibility appears pivotal to firearm safety messaging and programming. Pairing locally trusted messengers like law enforcement with familiar community organizations like FFA and 4-H may increase engagement with safe firearm storage content. Given the mixed views on device incentives, programs should consider prioritizing demonstrations and practical options (lockboxes or safes) while avoiding one-size-fits-all approaches. Future research and program development should consider leveraging these trusted sources to enhance the reach and acceptance of firearm safety interventions in rural communities.

This study has several potential limitations. First, the number of participants in the focus groups was relatively small. Although data saturation was achieved for the research questions, the limited sample size should be considered when interpreting the findings. Second, the study was conducted exclusively with rural parents who farm/ranch in Iowa which may limit the generalizability of the results to rural populations in other U.S. states or communities with more diverse races/ethnicities. Third, social desirability bias may have occurred when reporting storage practices in the focus group setting. Fourth, parents who choose to attend a safety focus group may differ from those who do not, potentially limiting the transferability of the findings. Fifth, most study participants were female, whereas firearm ownership is more common among males in rural areas [33, 34], which may limit the transferability of the findings to other rural populations.

## Conclusions

When developing firearm safety messaging and programming, it is essential to consider the attitudes and perspectives of rural adults. Researchers must account for who is delivering the message and consider the content and the mode of delivery. These insights should be systematically incorporated into intervention designs to ensure that firearm safety initiatives are effectively adapted and implemented within rural, farming communities.
